# Simulation and prediction of changes in tree species composition in subtropical forests of China using a nonlinear difference equation system model

**DOI:** 10.3389/fpls.2023.1280126

**Published:** 2023-11-17

**Authors:** Biyong Ji, Kunyong Yu, Fan Wang, Hongli Ge, Jian Liu

**Affiliations:** ^1^ College of Forestry, Fujian Agriculture and Forestry University, Fuzhou, China; ^2^ Zhejiang Forest Resources Monitoring Center, Hangzhou, China; ^3^ University Key Lab for Geomatics Technology and Optimize Resource Utilization in Fujian Province, Fujian Agriculture and Forestry University, Fuzhou, China; ^4^ College of Environment and Resources Science, Zhejiang Agriculture and Forestry University, Hangzhou, China

**Keywords:** species composition, species abundance, species abundance limit value, forest succession, nonlinear difference equation system (NDES) model, subtropical forest

## Abstract

Changes in tree species composition are one of the key aspects of forest succession. In recent decades, significant changes have occurred in the tree species composition of subtropical forests in China, with a decrease in coniferous trees and an increase in broad-leaved trees. This study focuses on Zhejiang Province, located in the subtropical region of China, and utilizes seven inventories from the National Continuous Forest Inventory (NCFI) System spanning 30 years (1989-2019) for modeling and analysis. We categorized tree species into three groups: pine, fir, and broadleaf. We used the proportion of biomass in a sample plot as a measure of the relative abundance of each tree species group. A novel nonlinear difference equation system (NDES) model was proposed. A NDES model was established based on two consecutive survey datasets. A total of six models were established in this study. The results indicated that during the first two re-examination periods (1989-1994, 1994-1999), there was significant fluctuation in the trend of tree species abundance, with no consistent pattern of change. During the latter four re-examination periods (1999-2004, 2004-2009, 2009-2014, 2014-2019), a consistent trend was observed, whereby the abundance of the pine group and the fir group decreased while the abundance of the broad-leaved group increased. Moreover, over time, this pattern became increasingly stable. Although the abundances of the pine group and the fir group have been steadily declining, neither group is expected to become extinct. The NDES model not only facilitates short-term, medium-term, and even long-term predictions but also employs limit analysis to reveal currently obscure changing trends in tree species composition.

## 1 Introduction

As an essential part of the global ecosystem, the forest ecosystem is a complex heterogeneous hierarchical system characterized by dynamic species replacement (Bruelheide et al., 2011). Forest succession can reveal changes in the whole forest, affect the wood supply of the forest (Lantz et al., 2022), and influence ecological functions, such as the supply of ecosystem services (Cortés-Calderón et al., 2021), carbon sequestration (Dulamsuren, 2021), soil carbon accumulation *(* Zhu et al., 2021), and animal and plant habitation (González et al., 2022). Species composition is a vital expression of forest succession (Mi et al., 2016). Species composition can reflect the composition of tree species in forest ecosystems and the reasonable proportion of each tree species (He et al., 2015; Pelissari et al., 2017). It is necessary to characterize the changes in species composition and predict the forest succession process (Tian et al., 2022), which helps us better understand the effects of human disturbance and climate change on species composition and provide sustainable forest management (Rutishauser et al., 2015).

Mathematical models have been widely used to simulate and analyze the process of forest succession. There is a category known as forest gap models, which primarily focus on small-scale forest succession. The patterns of forest succession, such as the optimal growth, replacement, and death of a single tree or the gap phase dynamics at the plot scale, can be well demonstrated using the succession gap model (Larocque and Bell, 2021). Examples of these models include JABOWA (Botkin et al., 1972), FORET(Shugart and West, 1977; Bormann and Likens, 1979), SORTIE (Pacala et al., 1993; Bugmann, 2001), and FAREAST (Yan and Shugart, 2005). Some of researchers prefer highly parametric models to simulate the photosynthesis and transpiration of individual leaves, as these models can well explain the process of dynamic forest succession (Medlyn et al., 2005; Ibrom et al., 2006). In addition, climate-sensitive gap models were applied to capture the effects of climate and ecological processes on future forest dynamics, such as ForClim (Mina et al., 2015; Martin-Benito et al., 2022), TreeMig (Thuiller et al., 2008), FireBGCv2 (Keane et al., 2011), PnET-Succession(Scheller et al., 2011), and FAREAST (Hu et al., 2018).

With increasing simulation scale, stand-level and landscape-level models are being proposed in forest succession research. For example, LANDIS-II (De Jager et al., 2019), FATES (Koven et al., 2020), FORMIND (Rödig et al., 2017) and SIMREG (Perin et al., 2021) have been applied to simulate the formation, development, disturbance, degradation, and other processes of forests on a wide range of spatial and temporal scales. Some of these researchers in this area have paid more attention to the basal area and compositional changes during tropical forest succession, and demographic forest models have been developed to analyze the growth-survival and stature-recruitment trade-offs (Ouyang et al., 2016; Hu et al., 2018; Ruger et al., 2020). Other researchers have focused on the dynamic disturbances involved, such as the survival rate of tree species in the process of forest succession (Peng et al., 2010), the regeneration rate after disturbance (Blanco et al., 2021), and the occurrence of natural disasters (Yin et al., 2009). The Markov chain model is used to describe the state of the forest ecosystem in forest growth scenarios, in which dynamic disturbances are treated as special events of forest growth scenarios that are constantly transferred according to a certain probability (Risch et al., 2009). Ecological process models are commonly employed for predicting changes in forest tree species composition, such as forecasting the loss and replacement of 111 tree species in China under climate change (Li et al., 2020).

In terms of research content, some researchers have emphatically explored the vertical structure changes of subtropical tree species (Yang et al., 2014), community structure changes (Xu et al., 2020), phylogenetic turnover (Chen et al., 2017; Michalski et al., 2017; Zhang et al., 2021), and ecological memory (Sun et al., 2013). Some researchers are concerned about how interacting forest components affect forest succession (Norden et al., 2015).

Most of the current research on forest succession is based on time series data (Clara et al., 2018; Mathieu et al., 2022). Studies have shown that difference equation theory can provide novel insights into forestry (Tomé et al., 2006; Chen, 2021). For example, Gilbertson and Kot (2021) proposed applying the Pioneer-Climax model based on difference equations to conduct a nine-case steady-state analysis of the long-term behavior of pioneer and climax species. Fort and Grigera (2021) developed a logistic difference model based on linear Lotka-Volterra equations to model and predict the tree species composition of the Barro Colorado tropical forest. Utilizing national forest resource continuous inventory data provides numerous conveniences for simulating and predicting forest tree species composition. For instance, Nikolova et al. (2019) employed generalized linear models based on forest inventory data from the National Continuous Forest Inventory (NCFI) to simulate and predict tree species composition in Swiss forests.

The tree species in Zhejiang Province effectively represent the forest ecosystems of South China and East China (Wu, 1980; Zhang et al., 2022). The distributed forests of Masson pine (*Pinus massoniana*), mixed conifer-broadleaf forests, and broad-leaved forests within the region represent different stages of forest succession, reflecting the temporal sequence of tree species composition evolution in the subtropical region of China (Sun et al., 2013; Li et al., 2021; Zhang et al., 2011; Piao et al., 2015).

Over the past few decades, due to sustained economic development and increased awareness of ecological benefits, as well as the impact of pine wilt disease (Hao et al., 2022) and a decline in timber prices, the forest species composition in Zhejiang Province has undergone significant changes. The proportion of pine and fir tree species has consistently decreased, while the broadleaf tree species have notably increased (Lin et al., 2012). This shift is not unique to Zhejiang Province but is observed across much of the subtropical regions in China. Simulating and analyzing this change and forecasting its trends is evidently crucial and meaningful. Where will this transformation ultimately lead? Will pine and fir species become extinct? To date, there is limited reporting on macro-temporal studies of tree species composition changes in the subtropical regions of China. This study utilized NCFI data from Zhejiang Province to investigate the relationship and dynamics among three major tree species groups: pines, firs, and broadleaves. The tree species group abundance (referred to as species abundance in some cases) was quantified using the proportion of biomass in each plot. Based on the plot-level species abundance, a nonlinear difference equation system (NDES) model comprising three equations was developed to simulate the temporal changes in tree species composition over the past 30 years (1989-2019) in Zhejiang Province. Furthermore, long-term trends and ultimate scenarios (limits) of future changes were analyzed.

## 2 Materials and methods

### 2.1 Study area

**Figure 1:**
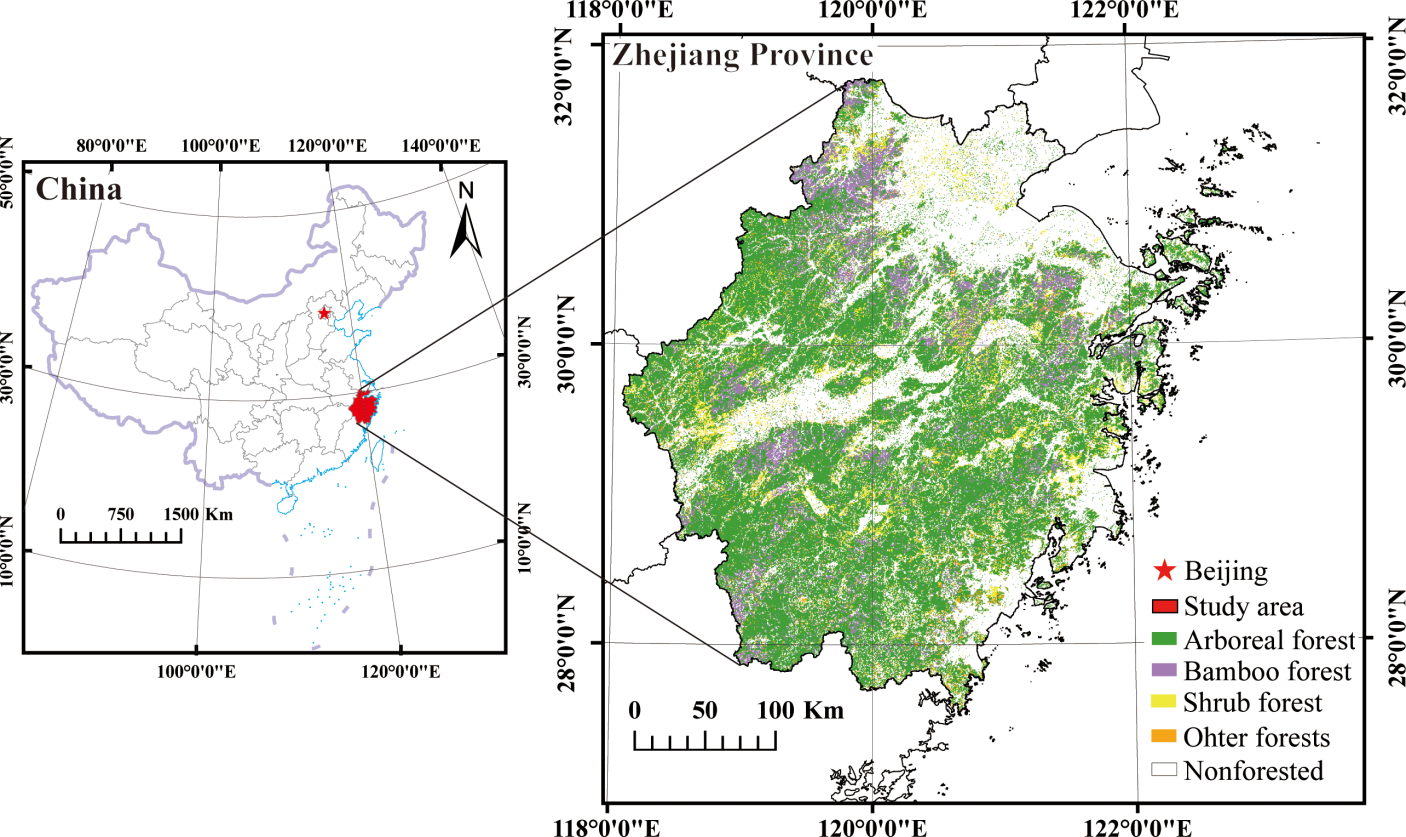
The distribution of forest resources in Zhejiang Province, China. (Note:Image cited from:http://bzdt.ch.mnr.gov.cn/browse.html?picId=%224o28b0625501ad13015501ad2bfc0290%22.).

Zhejiang Province is located along the southeastern coast of mainland China (118°01’ to 123°10’ E, 27°02’ to 31°11’ N) (**Figure 1**). The region belongs to the subtropical monsoon humid climate zone. The annual average temperature ranges from 16.1 to 18.6°C, and the annual average precipitation ranges from 1109.1 to 2132.3 millimeters. The southwestern part of the region is characterized by higher elevation, while the northeastern part is relatively lower. The total land area is approximately 105,500 square kilometers, with approximately 70% consisting of mountains and hills. Plains are predominantly distributed in the northern region. In 2020, the forest area in the study area was 60,800 square kilometers, accounting for 61.17% of the total land area in the province. The vegetation in the region is characterized by zonal vegetation, predominantly consisting of subtropical evergreen broad-leaved forests. The main broad-leaved tree species include *Quercus* L, *Cinnamomum camphora*, and *Schima superba* Gardn. et Champ. The main coniferous tree species include *Pinus massoniana*, *Pinus elliottii*, *Pinus taiwanensis* Hayata, and *Cunninghamia lanceolate* (Tao et al., 2022).

### 2.2 Experimental design and data preprocessing

**Figure 2:**
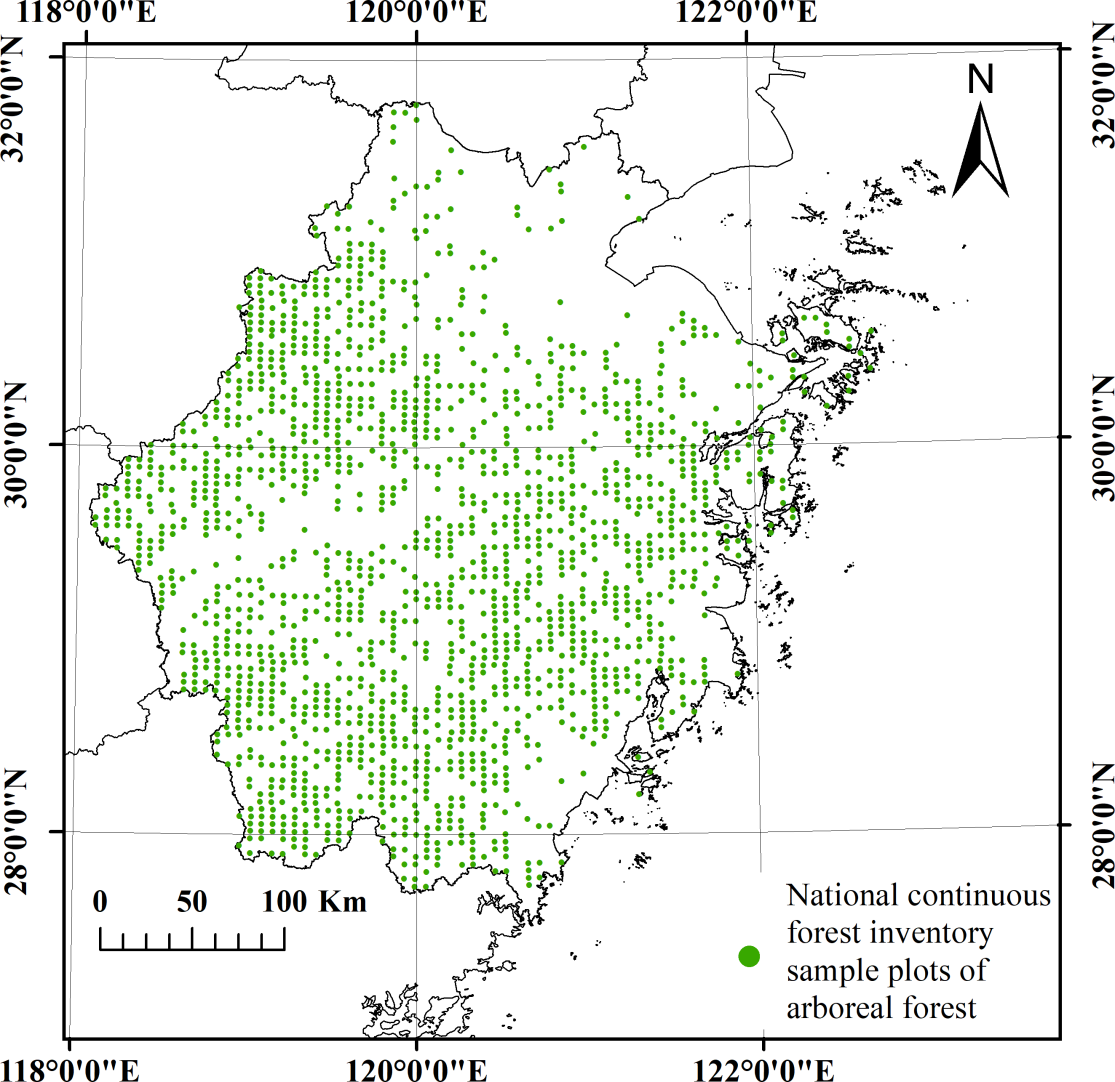
Distribution of NCFI plots in Zhejiang Province, China.

The data were collected from forest plots within the permanent plots of the NCFI in Zhejiang Province, China (**Figure 2**). A total of 4250 permanent plots were established across the entire province using a grid of 4 km × 6 km. The plots were square with an area of 800 square meters. Individual measurements were conducted for all trees within the plots with a diameter at breast height (DBH) equal to or greater than 5 centimeters (NFGA, 2020). The survey interval for NCFI was 5 years. The NCFI data selected for analysis were obtained from surveys conducted in 1989, 1994, 1999, 2004, 2009, 2014, and 2019, spanning a total period of 30 years. Subsequently, the entire set of seven datasets was divided into six periods: 1989-1994, 1994-1999, 1999-2004, 2004-2009, 2009-2014, and 2014-2019. The plots within the same period were paired. The plots were required to be forested at the beginning of the reexamination period (early stage).

In forest management practices in subtropical China, tree species are often categorized into three groups: pine, fir, and broadleaf. Such classification meets the requirements of general forest management practices. Classifying tree species is crucial for determining dominant tree species within a community (Kazmierczak et al., 2014). The pine group includes *Pinus massoniana* Lamb, *Pinus thunbergii* Parl, *Pinus taiwanensis* Hayata, *Pinus elliottii* Engelm, and others. Among them, *Pinus massoniana* Lamb (Masson pine) accounts for the vast majority. The fir group includes *Cunninghamia lanceolata* (Lamb.) Hook, *Cryptomeria fortune* Hooibrenk ex Otto et Dietr, *Metasequoia glyptostroboides* Hu & W. C. Cheng, *Taxodium ascendens* Brongn, and *Taxus wallichiana* var. chinensis (Pilg.) Florin, and others. Among them, *Cunninghamia lanceolata* (Lamb.) Hook (Chinese fir) constitutes the vast majority. The broad-leaved group includes *Quercus*, *Castanopsis*, *Liquidambar*, *Schima superba* Gardn. et Champ., Lauraceae (Sun et al., 2020), and others.

**Figure 3:**
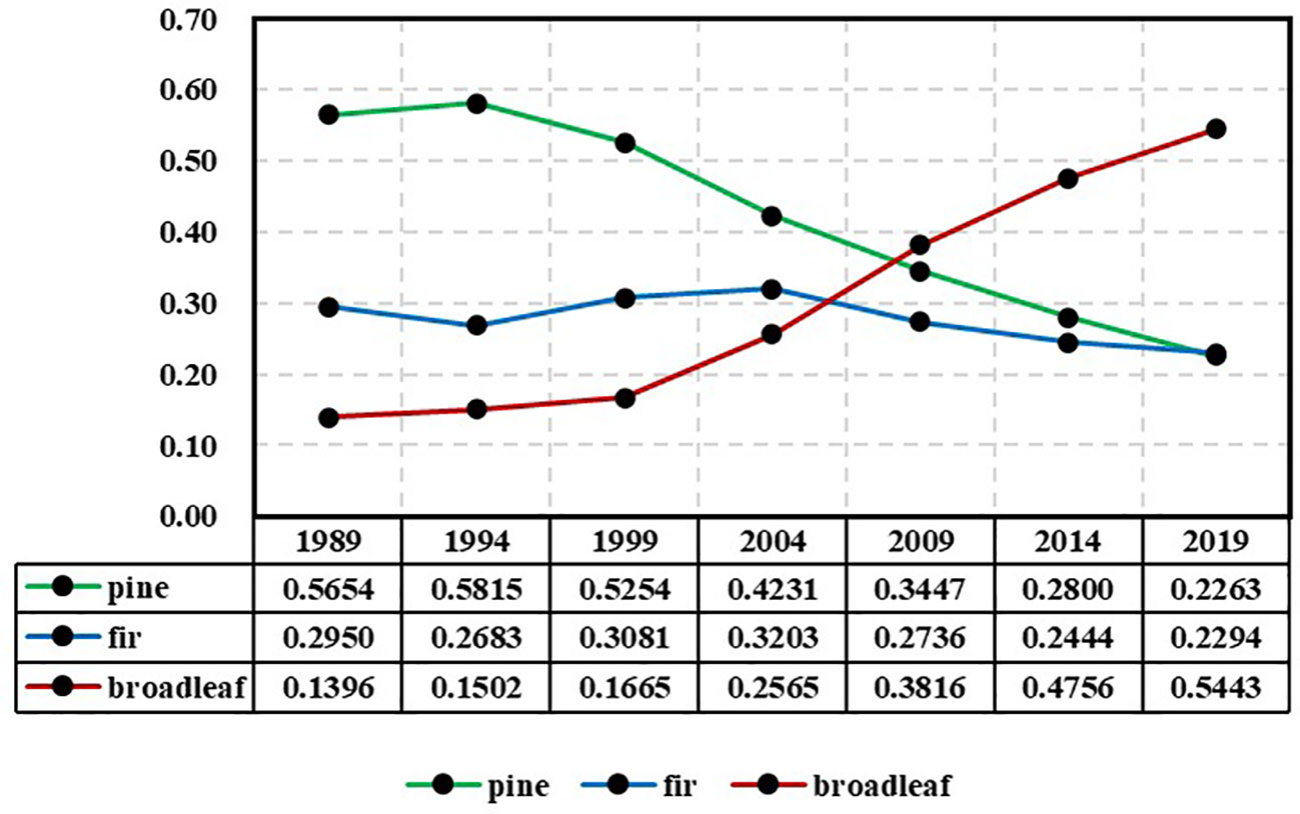
Changes in the average abundance of each species group (pine, fir, and broadleaf) during 1989-2019 based on the CFI data.

**Table 1** displays the number of remeasured forest plots in each period based on dominant tree species group statistics. The tree species group that exhibited the highest proportion of biomass within a plot was considered the dominant tree species group. The forest plots in the first three periods were relatively fewer compared to the latter three periods because in the surveys conducted in 1994, 1999, and 2004, one-third of the plots were relocated and could not be paired. The tree biomass was calculated based on plot stem measurement data (Ji et al., 2012). The biomass proportion of each tree species group in each plot was calculated as an indicator of species group abundance, where the sum of abundance indices for the three tree species groups within each plot was equal to 1. The variation in actual tree species abundance across years is illustrated in **Figure 3**, revealing significant trends. The pine group exhibits a continuous decline, transitioning from absolute dominance to absolute inferiority, whereas the broad-leaved group exhibits the opposite pattern.

**Table 1:** The numbers of plots in each reexamination period.

Amounts of plots	1989-1994	1994-1999	1999-2004	2004-2009	2009-2014	2014-2019
Total	842	824	909	1582	1657	1712
pine dominant	493	498	481	683	575	478
fir dominant	236	211	294	483	432	402
broadleaf dominant	113	115	134	416	650	832

A group-dominant plot is dominated by one group of trees species with the largest proportion of the gross biomass.

### 2.3 Nonlinear difference equation system model

In this study, a nonlinear difference equation system (NDES) model was developed, consisting of three difference equations (the model construction and derivation process are described in Appendix A). The NDES model is presented as follows:


(1) $$\{\begin{array}{c} y_{11}=\frac{1}{1+t_{1}e^{t_{3}y_{10}+t_{4}y_{20}}+t_{2}e^{t_{5}y_{10}+t_{6}y_{20}}}\equiv f_{1}\;\\ y_{21}=\frac{1}{(1/t_{1})e^{-t_{3}y_{10}-t_{4}y_{20}}+1+(t_{2}/t_{1})e^{(t_{5}-t_{3})y_{10}+(t_{6}-t_{4})y_{20}}}\equiv f_{2}\;\\ y_{31}=\frac{1}{(1/t_{2})e^{-t_{5}y_{10}-t_{6}y_{20}}+(t_{1}/t_{2})e^{(t_{3}-t_{5})y_{10}+(t_{4}-t_{6})y_{20}}+1}\equiv f_{3}\; \end{array}$$

where *y_ij_* represents the species abundance, *i* denotes the species group, i=1 (pine), 2 (fir), 3 (broadleaf), and *j* represents the temporal sequence of the remeasured plots, *j*=0 (former), 1 (latter). Auto conform 0≤*y_ij_ ≤* 1, *y*_11_+*y*_21_+*y*_31 = _1. *t*_1_, *t*_2_, *t*_3_, *t*_4_, *t*_5_, and *t*_6_ are 6 parameters of the NDES model.

Rewriting Eq. (1) in vector form:


(2) $$Y_{1}=F(Y_{0})$$

where $Y_{1}={\left(y_{11},y_{21},y_{31}\right)}^{'}$ , $Y_{0}={\left(y_{10},y_{20},y_{30}\right)}^{'}$ , and $F={\left(f_{1},f_{2},f_{3}\right)}^{'}$ . By substituting *Y*_0_ into the right side of Eq. (2), the estimated value *Y*_1_ for 5 years later (the remeasurement interval in this study is 5 years) can be obtained. Similarly, by substituting *Y*_1_ into the right side of Eq. (2), the estimated value *Y*_2_ for 10 years later can be obtained. This process continues iteratively, such that if *Y_k_* (*k*=0,1,2,…) is already known, *Y_k_*_+1_ can be calculated as *Y_k_*_+1_=*F*(*Y_k_*).

The estimation of the model parameters is based on plot-level data. For each plot, the species abundance (*y*_ij_) is calculated, including both the early and late stages. First, the total biomass of the plot is computed, and the proportion of biomass for each species group to the total biomass represents the species abundance of that particular species group.

### 2.4 Prediction of species abundance limits, interval analysis and hypothesis

If $\underset{k\to \infty }{\lim}F(Y_{k})=Y^{*}$ , i.e., $Y^{*}=F(Y^{*})$ , where $Y^{*}={\left(y_{1}^{*},y_{2}^{*},y_{3}^{*}\right)}^{'}$ is a constant vector, then $Y^{*}$ is referred to as the equilibrium point of the difference equation system, and such an equilibrium point is inherently stable (Elaydi, 2005). A model with a stable equilibrium point possesses significant biological significance, as it facilitates theoretical analysis and long-term predictions. In this study, the equilibrium point is referred to as the limit, representing the theoretical ultimate state of tree species abundance variations.

Reliability analysis is necessary for limits. This paper simulates the variance of limit $Y^{*}$ through Monte Carlo methods. Let $T={\left(t_{1},\cdots ,t_{6}\right)}^{'}$ be the estimated value of the model parameter, with its covariance matrix denoted as $\sum^{}$ . Let $T^{0}∼N\left(T,{\;}^{\sum }\right)$ , and generate a set of random numbers $T^{0}={\left(t_{1}^{0},\cdots ,t_{6}^{0}\right)}^{'}$ for each plot. Taking the latter observed abundance of the plot as the initial value, we calculated the limit of the model by considering *T* ^0^ as the model parameter. We estimate the covariance matrix $D(Y^{*})$ by computing the covariance matrix $\Sigma_{3\times 3}^{*}$ based on the simulated limits of all *n* plots and consider it as an estimate for $D(Y^{*})$ . Since $y_{1}^{*}+y_{2}^{*}+y_{3}^{*}=1$ , the sum of the elements in matrix $\Sigma_{3\times 3}^{*}$ is zero.

Interval estimation of the abundance limit for a single tree species group. Assume that the abundance limit follows a normal distribution. Because the abundance limit is bounded between 0 and 1, it is theoretically appropriate to use a truncated normal distribution. Let $y^{*}$ be the point estimate of the abundance limit for a certain tree species group and $\sigma^{2}$ be the variance of $y^{*}$ , represented by the diagonal elements in matrix

$\Sigma_{3\times 3}^{*}$ . Let $p(y,y^{*},\sigma^{2})$ denote the probability density function of the normal distribution. The probability density function of the truncated normal distribution is given by:


(3) $$p_{\text{tr}}(y,y^{*},\sigma^{2})=p(y,y^{*},\sigma^{2})/\tau \;,\;\tau =\int_{0}^{1}p(y,y^{*},\sigma^{2})dy$$

Overall hypothesis testing of the limit vector. The overall hypothesis testing of the limit vector for tree species abundance was conducted using a multivariate normal distribution. Let $Y^{*}={\left(y_{1}^{*},y_{2}^{*},y_{3}^{*}\right)}^{'}$ be the point estimate of the limit vector and $Y^{0*}={\left(y_{1}^{0*},y_{2}^{0*},y_{3}^{0*}\right)}^{'}$ be another vector. The aim is to assess the possibility of $Y^{*}=Y^{0*}$ . The sum of the three components of the abundance limit is 1, implying that it has 2 degrees of freedom (3-1 = 2). Consequently, the rank of matrix $\Sigma_{3\times 3}^{*}$ is also 2, indicating that the matrix is not full rank and thus cannot be inverted. Considering the abundance limits of the first two tree species groups, namely, pines and firs, the determination of the abundance limit for broadleaves was subsequently established. Therefore, we only need to examine the limits of the first two tree species groups.

Let $Y^{*(2)}={\left(y_{1}^{*},y_{2}^{*}\right)}^{'}$ , $Y^{0*(2)}={\left(y_{1}^{0*},y_{2}^{0*}\right)}^{'}$ and $\Sigma_{2\times 2}^{*(2)}=\left(\begin{array}{cc} \sigma_{1}^{2} & \sigma_{1\cdot 2}\\ \sigma_{2\cdot 1} & \sigma_{2}^{2} \end{array}\right)$ , and compute the F-statistic:


(4) $$F=\frac{\left(n-p\right)}{(n-1)p}{\left(Y^{*(2)}-Y^{0*(2)}\right)}^{'}{\left(\Sigma_{2\times 2}^{*(2)}\right)}^{-1}(Y^{*(2)}-Y^{0*(2)})∼F(p,n-p)$$

The F distribution has the first degree of freedom equal to *p* and the second degree of freedom equal to *n*-*p*, where *p* is 2. To test whether both pines and firs may converge to zero in the future, a hypothesis test can be conducted by setting $Y^{0*(2)}={\left(0,0\right)}^{'}$ .

### 2.5 Long-term prediction of tree species abundance

To uniformly forecast tree species abundance until 2119, a comparison of the prediction curves from models of different periods was enabled. This analysis can then be used to examine the changes in tree species composition over the past 30 years within the modeling dataset. Based on the plot data and using the initial species abundance from the latter observations of the modeling dataset, iterative calculations can be performed to obtain predicted values of species abundance for every 5-year interval in the plots. The average of the predicted species abundance values for the plots was calculated as the predicted average value of species abundance for the entire province. A predicted value for a plot is dependent only on the previous adjacent state and is not influenced by other preceding states (Scherstjanoi et al., 2014).

## 3 Results

### 3.1 Model parameter estimation results and the actual prediction accuracy

The parameters of the nonlinear differential equation model in each reexamination period are shown in **Table 2**. The parameters had a certain regularity. t1 ranged from 0.722 to 1.5111, t2 ranged from 20.4263 to 42.8872, t3 ranged from -3.3836 to -2.3495, t4 ranged from 2.5992 to 3.5865, t5 ranged from -6.9519 to -5.4936, and t6 ranged from -4.4867 to -2.8888 (**Table 2**). The same parameter in different reexamination periods had the same trend label, i.e., positive or negative. The coefficients of determination were greater than 0.8.

**Table 2:** The parameters of the nonlinear differential equation model in each reexamination period.

Parameter	1989-1994	1994-1999	1999-2004	2004-2009	2009-2014	2014-2019
*t*_1_	0.7220	1.0682	0.8203	1.3904	1.1641	1.5110
*t*_2_	35.8226	20.4263	32.2185	42.8872	32.1222	33.1472
*t*_3_	-2.8635	-2.7597	-2.3495	-3.1068	-2.9822	-3.3836
*t*_4_	3.5865	2.7024	3.1429	2.5992	2.8834	2.8001
*t*_5_	-6.9519	-5.7101	-5.6925	-5.8867	-5.4936	-5.5520
*t*_6_	-4.4867	-3.8109	-3.3400	-3.1080	-2.9301	-2.8888
$R_{1}^{2}$	0.9803	0.9203	0.9123	0.9298	0.9118	0.9117
$R_{2}^{2}$	0.9695	0.8727	0.9150	0.9070	0.9071	0.9095
$R_{3}^{2}$	0.9516	0.8180	0.8156	0.9014	0.9387	0.9581

Note that $t_{1},t_{2},t_{3},t_{4},t_{5},t_{6}$ are the 6 parameters of the model in six reexamination periods, and $R_{1}^{2}$ , $R_{2}^{2}$ $R_{3}^{2}$ represent the coefficients of determination of these three groups of tree species, pine, fir and broadleaf respectively.

The predicted data are shown in **Table 2**. The abundance data in the table represent predicted values. The relative error is defined as the difference between the predicted values and actual values divided by the actual values. The actual values are shown in **Figure 3**. In general, as the prediction horizon increases, the error also increases. However, for the same prediction horizon, the models in the first two periods (1989-1994 and 1994-1999) exhibit significantly larger prediction errors compared to the models in the subsequent four periods.

### 3.2 Prediction of species abundance limit

**Figure 4:**
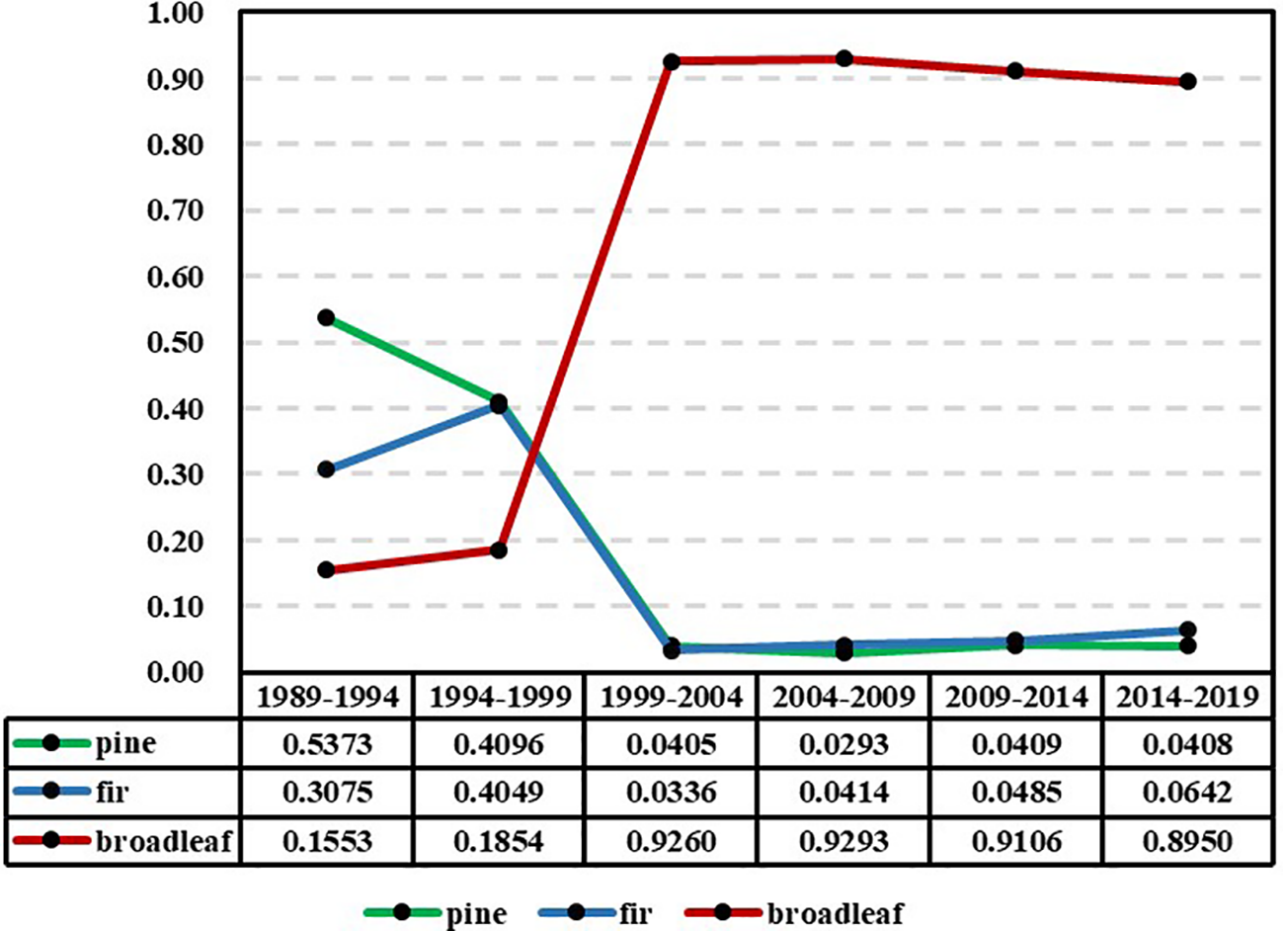
Estimation results of the limit value of the abundance of the pine, fir, and broad-leaved groups in each reexamination period (y-axis: the limit value of the abundance of the species groups).

To analyze the ultimate trend of the models in each period, the models were subjected to limit prediction. **Figure 4** displays the predicted limit values of species abundance for each period model. It is evident that the timeline can be divided into two distinct phases, with 1999 as the dividing point: two periods prior to 1999 (1989-1994 and 1994-1999) and four periods following 1999 (1999-2004, 2004-2009, 2009-2014, and 2014-2019). The former phase exhibits significant fluctuations, whereas the latter phase shows minimal variations.

### 3.3 Long-term prediction of species abundance

**Figure 5:**
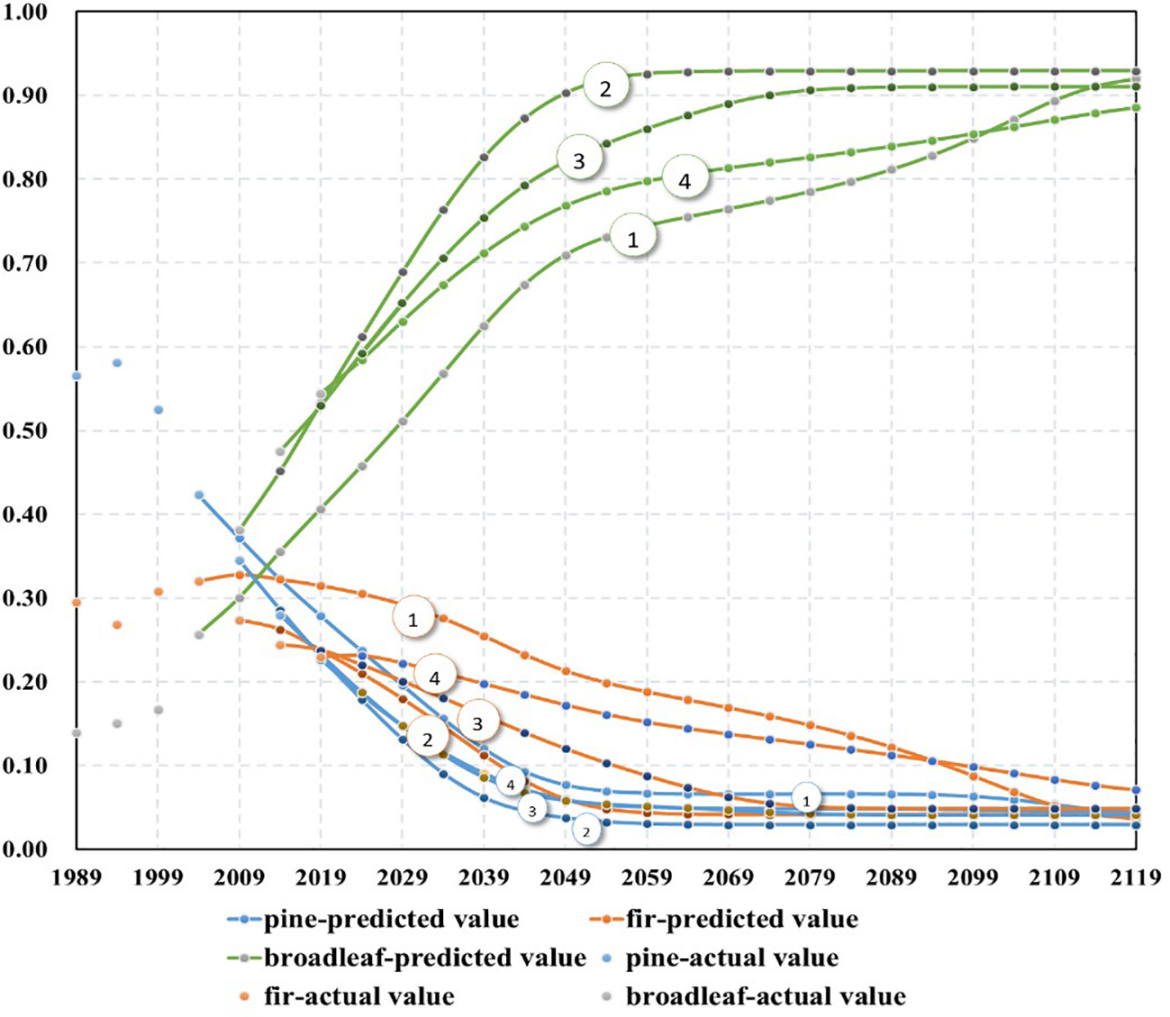
The prediction of abundance of species groups in different reexamination periods (model 1: model based on the reexamination period, 1999-2004; model 2: model based on the reexamination period, 2004-2009; model 3: model based on the reexamination period, 2009-2014; model 4: model based on the reexamination period, 2014-2019).

Based on the preceding limit analysis, it is evident that the trends of the models in the latter four periods are similar. In other words, over the past 20 years, the changes in species abundance have exhibited a consistent pattern. Now, a comparative analysis of long-term predictions is conducted based on the models from these four periods. Each period model predicts until the year 2119, with the shortest prediction spanning 100 years (2019-2119) and the longest prediction spanning 115 years (2004-2119) (**Figure 5**). Based on the predicted trends, there is a significant decrease in the abundance of pines, a decrease in firs, and a noticeable increase in broadleaves. This trend is consistent with the field CFI data. The trend lines of species abundance share a common characteristic: lines ① and ② are the furthest apart, while lines ③ and ④ are closest to each other and sandwiched between lines ① and ②. This indicates that the two models from the first 10 years exhibit greater fluctuations compared to the two models from the latter 10 years. The patterns of species abundance have become increasingly stable over the past 20 years.

### 3.4 Interval estimation and hypothesis testing

The interval estimation and hypothesis testing procedures for the limit values of each period model are identical. Take the most recent period (2014-2019) as an example. In our study, the predicted values of the species abundance of broad-leaved trees fluctuated significantly with the use of model one and model two, while the predicted curves fluctuated less between model three and model four. In addition, the difference in the predicted species abundance of broad-leaved trees was the lowest (gray line one) when model one was used. The number of plots, *n*, is 1712. The model parameter *T* can be found in the last column of **Table 3**. **Table 4** presents the interval estimation results of species abundance for pines, firs, and broadleaves based on the 2014-2019 model obtained using the truncated normal distribution method.

**Table 3:** Comparison of relative prediction error of abundance in different reexamination periods.

The reexamination periods	Predicting year	Abundance of the pine group	Abundance of the fir group	Abundance of the broad-leaved group	Error in abundance of the pine group (%)		Error in Abundance of the fir group (%)	Error in Abundance of the broad-leaved group (%)
1989-1994	1999	0.5623	0.2770	0.1607		7.03	-10.10	-3.49
	2004	0.5564	0.2801	0.1636		31.48	-12.57	-36.24
	2009	0.5542	0.2810	0.1648		60.76	2.67	-56.81
	2014	0.5534	0.2810	0.1656		97.64	14.98	-65.18
	2019	0.5530	0.2809	0.1661		144.33	22.47	-69.48
1994-1999	2004	0.4695	0.3461	0.1845		10.95	8.03	-28.10
	2009	0.4520	0.3571	0.1908		31.12	30.51	-49.99
	2014	0.4402	0.3666	0.1932		57.24	49.97	-59.38
	2019	0.4315	0.3747	0.1938		90.66	63.38	-64.40
1999-2004	2009	0.3537	0.3079	0.3384		2.58	12.53	-11.32
	2014	0.3065	0.3011	0.3924		9.46	23.20	-17.49
	2019	0.2643	0.2926	0.4431		16.79	27.58	-18.60
2004-2009	2014	0.2768	0.2487	0.4745		-1.15	1.77	-0.23
	2019	0.2221	0.2250	0.5529		-1.88	-1.89	1.58
2009-2014	2019	0.2295	0.2317	0.5388		1.42	1.02	-1.02

Note that the abundance data are the predicted value, and the error is the relative error between the predicted value and the actual value. A positive number means that the predicted value is larger than the actual value, while a negative number means that the predicted value is smaller.

**Table 4:** The interval estimation results of the abundance for a single tree species group.

Species group	0.95%		0.99%	
	Lower limit	Upper limit	Lower limit	Upper limit
Pine	0.0354	0.0461	0.0337	0.0478
Fir	0.0561	0.0723	0.0536	0.0749
Broadleaf	0.8847	0.9053	0.8815	0.9086

The limit vector hypothesis testing results obtained using Eq. (4) are presented in **Table 5**. Hypothesis 1: $Y^{0*}$ represents the trend of the abundance of pines approaching zero, while there is no change in the abundance of firs; Hypothesis 2: $Y^{0*}$ represents the trend of the abundance of firs approaching zero, while there is no change in the abundance of pines; Hypothesis 3: $Y^{0*}$ represents the trend of the species abundance of both pines and firs approaching zero. The results indicate that none of these three hypotheses can be established. This indicates that despite both pines and firs tending toward absolute disadvantage, their species abundance cannot approach zero (i.e., extinction).

**Table 5:** Results of hypothesis testing of the abundance in three tree species groups.

Species group	$Y_{\infty }$	$Y_{\infty }^{0}$ (1)	$Y_{\infty }^{0}$ (2)	$Y_{\infty }^{0}$ (3)
Pine	0.0408	0	0.0408	0
Fir	0.0642	0.0642	0	0
Broadleaf	0.8950	0.9358	0.9592	1
*F*		112.54	122.95	204.56
*Sig*		0.000000	0.000000	0.000000

Note that there are three scenarios (1) (1) represents scenario one, where the abundance of the pine group tends to zero and the fir group $Y_{\infty }^{0}$ (2)does not change(2); $Y_{\infty }^{0}$ represents scenario two, where the abundance of the fir group tends to zero and the pine group (3)does not change(3); $Y_{\infty }^{0}$ represents scenario three, where the abundances of both the pine and the fir groups tend to zero.

## 4 Discussion

### 4.1 Through limit analysis, hidden patterns of variation can be revealed

The NDES model is developed based on the data of repeated measurements of permanent plots. Therefore, the variation trend in tree species abundance during the period of data collection determines the overall trajectory of the model. Under the assumption that the forest continues to develop according to the current trend, the model is expected to provide stable and reliable predictive performance. When the forest deviates from the current trend of development, the model’s predictions will deviate from the actual observations. This behavior is observed in nearly all models. A good model is capable of consistently forecasting this trend forward during predictions without experiencing significant biases solely due to extrapolation. From **Table 2**, it can be observed that for the same prediction timeframe, the models during the first two periods (1989-1994 and 1994-1999) exhibit significantly larger prediction errors compared to the models during the subsequent four periods. This indicates that there may be distinct differences in the patterns of species abundance variation between the first two periods and the latter four periods. Consequently, the models developed for the first two periods are not suitable for predicting the latter four periods. During the latter four periods, the forest exhibits a relatively consistent pattern of variation. As a result, the models developed for this phase all yield favorable predictive outcomes (refer to **Table 2**, **Figure 5**).

If the forest continues to develop steadily in a certain trend and a model can consistently reflect this developmental trend, then the theoretical limits of the model should reasonably reflect this trend. The following discussion addresses this issue from the perspective of limits. From **Figure 3**, it can be observed that the actual species abundance exhibits gradual changes over time. The abundance of pines shows an absolute dominance starting in 1989, reaching its peak value (0.5810) in 1994 and subsequently declining to gradually become an absolute inferiority. For firs, the abundance fluctuated upward from 1989 to 2004, reaching its peak (0.3203) in 2004 and then decreasing, transitioning from moderate dominance to absolute inferiority. The abundance of broadleaves maintained a stable growth trend and surpassed that of both pines and firs in 2009, becoming dominant and expanding its dominance thereafter. Despite significant changes over the 30-year period, there are no abrupt shifts observed in between.

The trend of abundance limits depicted in **Figure 4** is distinct from the actual value trend shown in **Figure 3**. The limits represent the ultimate state of abundance, which differs from the current actual values and signifies the long-term trend of abundance. From **Figure 4**, it can be observed that the limit distribution can be divided into two distinct phases, with 1999 serving as the boundary. Before 1999, the limits of the models during the periods of 1989-1994 and 1994-1999 exhibit fluctuations. The limit for pines shows a significant decline but remains in clear dominance along with firs. The limit for broadleaves shows an upward trend, while the limit for firs demonstrates a relatively rapid increase. This indicates that during these two periods, there are distinct differences in the trends of species abundance, and a stable pattern of variation has not yet been established. After 1999, the limits of species abundance for the four periods of 1999-2004, 2004-2009, 2009-2014, and 2014-2019 demonstrated remarkable stability. Throughout these periods, broadleaves consistently maintain absolute dominance, while pines and firs consistently remain in a state of absolute inferiority. This indicates that over the course of the subsequent 20 years, although there have been significant changes in the actual species abundance, the trend of these changes has become stable and relatively consistent, remaining within the same overall pattern. It can be observed that through limit analysis using the NDES model, certain patterns of variation can be revealed that may not be readily apparent. The NDES model is capable of adequately simulating the trend of variation and maintaining this trend consistently during long-term predictions, as long as there are no significant changes in the trend of forest tree species abundance. Therefore, the risks associated with long-term prediction (extrapolation) are minimal.

The limit analysis reveals the ultimate state of tree species abundance variation, while the interval analysis of the limits indicates the potential range of variation for this ultimate state. Although **Figure 3** indicates a decreasing trend in the actual abundance of pines and firs, the predictive figures in **Figure 5** also demonstrate the same pattern. However, the interval analysis of the limits suggests that the probability of their abundance approaching zero is nearly zero. In other words, while pines and firs may perpetually remain in a state of absolute inferiority, they are highly unlikely to go extinct.

### 4.2 Comparison with existing methods

In terms of model classification, the NDES model proposed in this study is an empirical model, which fundamentally differs from the category of gap model and ecological process model. In the empirical modeling approach, the model proposed in this study possesses its own unique structure, which is entirely distinct from the models employed by Nikolova et al. (2019); Fort and Grigera (2021), and others.

In terms of the number of tree species (groups) that the model can simulate, the model proposed in this study is capable of simulating only three tree species (groups), which is significantly fewer compared to the models employed by Nikolova et al. (2019); Fort and Grigera (2021), and others. However, the model proposed in this study is designed to meet the practical needs of forest management in the subtropical forests of China. It is capable of fulfilling the application requirements in this specific context. From an ecological research perspective, the model’s results can provide a macroscopic understanding of the changes in tree species composition within a large-scale forest region. The number of difference equations in the model proposed in this study is equal to the number of tree species (groups) under investigation. It is evident that the model can be expanded according to the methods provided in Appendix A to meet the research requirements as needed.

The predicted tree species abundances from the NDES model are constrained to be nonnegative and cannot exceed 1. The sum of the abundances is guaranteed to be equal to 1. Such properties enhance the stability of the model. The limits of the NDES model effectively capture the trend of variation in the modeling data. Therefore, a key application of the NDES model is to predict potential future trends and ultimate states through limit analysis. It is a big characteristic of the proposed model. Currently, there is scarce literature reporting on such research.

### 4.3 Analysis of the driving forces behind changes in forest tree species abundance in Zhejiang Province

The primary forest plant community in Zhejiang Province is characterized by evergreen broad-leaved forests. However, prolonged anthropogenic activities have led to significant degradation of these forests in most areas, resulting in the emergence of substantial secondary forests, primarily dominated by Masson’s pine, and mixed coniferous and broad-leaved secondary forests. In recent decades, the province has experienced economic development, a reduced dependence on forest-based economies, and an increased awareness of forest conservation, which has facilitated rapid forest recovery. The decrease in coniferous tree populations is attributed to the prevalence of pine wilt disease and fluctuations in timber prices. This reduction in coniferous trees has created ecological opportunities for the growth of broad-leaved species. Broad-leaved trees typically exhibit greater shade tolerance compared to conifers, leading to an increase in forest density. Rising labor costs have resulted in reduced forest management intensity, further contributing to increased forest density. When forest density reaches a certain threshold, strong shade-intolerant tree species such as pines and firs are outcompeted by broad-leaved species, leading to increased mortality. Under these circumstances, even in the absence of other influencing factors, the proportion of coniferous trees is expected to decline. These factors have resulted in the forest ecosystem of Zhejiang Province undergoing a forest succession process that closely approximates a natural state. Therefore, over the past 20 years, the species composition of forests in Zhejiang Province has maintained a fundamentally similar trend and has become increasingly stable. Among the four predicted curves in **Figure 5** for the most recent four periods, curve ① from 1999-2004 and curve ② from 2004-2009 are far apart, while curve ③ from 2009-2014 and curve ④ from 2014-2019 are closer to each other, indicating a diminishing fluctuation between the curves. These circumstances indicate that over the past few decades, the changes in Zhejiang Province’s forests have primarily been driven by economic development and an increased awareness of conservation. The spread of pine wilt disease and fluctuations in timber prices have accelerated these changes. Once these changes reach a certain threshold, natural succession becomes the dominant driving force.

## 5 Conclusions

Due to various reasons, pine and fir tree species in Zhejiang Province have been consistently decreasing, and this trend is expected to persist over the long term. The fir group shifted from a relative advantage to an absolute disadvantage. They will perpetually remain at an absolute disadvantage but will never go extinct. As the Climax community in subtropical forests is characterized by evergreen broad-leaved forests, the observed changes in forest tree species composition over the past few decades conform to the natural succession patterns of subtropical forests.

The nonlinear difference equation system (NDES) model proposed in this paper is suitable for simulating and predicting the forest tree species composition in the macroscopic region of subtropical China. It not only enables short-term, medium-term, and even long-term predictions but also allows in-depth exploration of currently obscure trends and their stability in forest tree species composition through limit analysis of the model. The application of the model can provide a basis for the development of sustainable forest management measures.

## Data availability statement

The original contributions presented in the study are included in the article/supplementary material. Further inquiries can be directed to the corresponding author.

## Author contributions

BJ: Funding acquisition, Writing – original draft, Data curation, Investigation, Methodology. KY: Conceptualization, Formal Analysis, Writing – original draft. FW: Methodology, Validation, Writing – original draft. HG: Methodology, Validation, Writing – original draft. LJ: Funding acquisition, Writing – review & editing.

## Appendix A. Derivation process of the nonlinear difference equation model for the abundance of the tree species group

The base model of the abundance of the tree species groups is set as the following exponential model:

A-Eq. (1)
$$\{\begin{array}{c} y_{11}=a_{10}e^{a_{11}y_{10}+a_{12}y_{20}+a_{13}y_{30}}\\ y_{21}=a_{20}e^{a_{21}y_{10}+a_{22}y_{20}+a_{23}y_{30}}\\ y_{31}=a_{30}e^{a_{31}y_{10}+a_{32}y_{20}+a_{33}y_{30}} \end{array}$$

where *y_10_*, *y_20_*, and *y_30_* represent the early abundances of the pine, fir and broad-leaved groups, respectively; *y_11_*, *y_21_*, and *y_31_* represent the late stage; $y_{11}+y_{21}+y_{31}=1$ ; $y_{10}+y_{20}+y_{30}=1$ ; and 0≤*y_11_*, *y_21_*, *y_31_*, *y_10_*, *y_20_*, *y_30_ ≤* 1 must be satisfied. $a_{1}{\;}_{0},a_{11},a_{12},a_{13}$ , $a_{2}{\;}_{0},a_{21},a_{22},a_{23}$ , $a_{30},a_{31},a_{32},and\;a_{33}$ are the 12 parameters of the model. Since $\;y_{11}+y_{21}+y_{31}=1$ , that is:

$a_{10}e^{a_{11}y_{10}+a_{12}y_{20}+a_{13}y_{30}}+a_{20}e^{a_{21}y_{10}+a_{22}y_{20}+a_{23}y_{30}}+a_{30}e^{a_{31}y_{10}+a_{32}y_{20}+a_{33}y_{30}}=1$ must be satisfied, and Equation A-Eq. (1) is adjusted to a proportional model:

A-Eq. (2)
$$\{\begin{array}{l} y_{11}=\frac{a_{10}e^{a_{11}y_{10}+a_{12}y_{20}+a_{13}y_{30}}}{a_{10}e^{a_{11}y_{10}+a_{12}y_{20}+a_{13}y_{30}}+a_{20}e^{a_{21}y_{10}+a_{22}y_{20}+a_{23}y_{30}}+a_{30}e^{a_{31}y_{10}+a_{32}y_{20}+a_{33}y_{30}}}\\ y_{12}=\frac{a_{20}e^{a_{21}y_{10}+a_{22}y_{20}+a_{23}y_{30}}}{a_{10}e^{a_{11}y_{10}+a_{12}y_{20}+a_{13}y_{30}}+a_{20}e^{a_{21}y_{10}+a_{22}y_{20}+a_{23}y_{30}}+a_{30}e^{a_{31}y_{10}+a_{32}y_{20}+a_{33}y_{30}}}\\ y_{13}=\frac{a_{30}e^{a_{31}y_{10}+a_{32}y_{20}+a_{33}y_{30}}}{a_{10}e^{a_{11}y_{10}+a_{12}y_{20}+a_{13}y_{30}}+a_{20}e^{a_{21}y_{10}+a_{22}y_{20}+a_{23}y_{30}}+a_{30}e^{a_{31}y_{10}+a_{32}y_{20}+a_{33}y_{30}}} \end{array}$$

A-Eq. (2) is reduced to:

A-Eq. (3)
$$\{\begin{array}{l} y_{11}=\frac{1}{1+(a_{20}/a_{10})e^{(a_{21}-a_{11})y_{10}+(a_{22}-a_{12})y_{20}+(a_{23}-a_{13})y_{30}}+(a_{30}/a_{10})e^{(a_{31}-a_{11})y_{10}+(a_{32}-a_{12})y_{20}+(a_{33}-a_{13})y_{30}}}\\ y_{21}=\frac{1}{(a_{10}/a_{20})e^{(a_{11}-a_{21})y_{10}+(a_{12}-a_{22})y_{20}+(a_{13}-a_{23})y_{30}}+1+(a_{30}/a_{20})e^{(a_{31}-a_{21})y_{10}+(a_{32}-a_{22})y_{20}+(a_{33}-a_{23})y_{30}}}\\ y_{31}=\frac{1}{(a_{10}/a_{30})e^{(a_{11}-a_{31})y_{10}+(a_{12}-a_{32})y_{20}+(a_{13}-a_{33})y_{30}}+(a_{20}/a_{30})e^{(a_{21}-a_{31})y_{10}+(a_{22}-a_{32})y_{20}+(a_{23}-a_{33})y_{30}}+1} \end{array}$$

Let

A-Eq. (4)
$$\{\begin{array}{l} k_{1}=a_{20}/a_{10},\;k_{2}=a_{30}/a_{10},\;a_{30}/a_{20}=k_{2}/k_{1}\\ k_{3}=a_{21}-a_{11},\;k_{4}=a_{22}-a_{12},\;k_{5}=a_{23}-a_{13},\;\\ k_{6}=a_{31}-a_{11},\;k_{7}=a_{32}-a_{12},\;k_{8}=a_{33}-a_{13} \end{array}$$

According to Eq. (4), we can obtain:

A-Eq. (5)
$$\{\begin{array}{l} a_{11}-a_{21}=-k_{3},\;a_{12}-a_{22}=-k_{4},\;a_{13}-a_{23}=-k_{5}\\ a_{31}-a_{21}=(a_{31}-a_{11})-(a_{21}-a_{11})=k_{6}-k_{3}\\ a_{32}-a_{22}=(a_{32}-a_{12})-(a_{22}-a_{12})=k_{7}-k_{4}\\ a_{33}-a_{23}=(a_{33}-a_{13})-(a_{23}-a_{13})=k_{8}-k_{5}\\ a_{11}-a_{31}=-k_{6},\;a_{12}-a_{32}=-k_{7},\;a_{13}-a_{33}=-k_{8}\\ a_{21}-a_{31}=k_{3}-k_{6},\;a_{22}-a_{32}=k_{4}-k_{7},\;a_{23}-a_{33}=k_{5}-k_{8} \end{array}$$

Then, Eq. (3) can be rewritten as:

A-Eq. (6)
$$\{\begin{array}{l} \;y_{11}=\frac{1}{1+k_{1}e^{k_{3}y_{10}+k_{4}y_{20}+k_{5}y_{30}}+k_{2}e^{k_{6}y_{10}+k_{7}y_{20}+k_{8}y_{30}}}\\ y_{21}=\frac{1}{(1/k_{1})e^{-k_{3}y_{10}-k_{4}y_{20}-k_{5}y_{30}}+1+(k_{2}/k_{1})e^{(k_{6}-k_{3})y_{10}+(k_{7}-k_{4})y_{20}+(k_{8}-k_{5})y_{30}}}\\ y_{31}=\frac{1}{(1/k_{2})e^{-k_{6}y_{10}-k_{7}y_{20}-k_{8}y_{30}}+(k_{1}/k_{2})e^{(k_{3}-k_{6})y_{10}+(k_{4}-k_{7})y_{20}+(k_{5}-k_{8})y_{30}}+1} \end{array}$$

According to $y_{10}+y_{20}+y_{30}=1$ , the elimination of $y_{30}$ gives:

A-Eq. (7)
$$\{\begin{array}{l} \;y_{11}=\frac{1}{1+k_{1}e^{k_{5}+(k_{3}-k_{5})y_{10}+(k_{4}-k_{5})y_{20}}+k_{2}e^{k_{8}+(k_{6}-k_{8})y_{10}+(k_{7}-k_{8})y_{20}}}\\ y_{21}=\frac{1}{(1/k_{1})e^{-k_{5}+(k_{5}-k_{3})y_{10}+(k_{5}-k_{4})y_{20}}+1+(k_{2}/k_{1})e^{(k_{8}-k_{5})+(k_{6}-k_{3}-k_{8}+k_{5})y_{10}+(k_{7}-k_{4}-k_{8}+k_{5})y_{20}}}\\ y_{31}=\frac{1}{(1/k_{2})e^{-k_{8}+(k_{8}-k_{6})y_{10}+(k_{8}-k_{7})y_{20}}+(k_{1}/k_{2})e^{(k_{5}-k_{8})+(k_{3}-k_{6}-k_{5}+k_{8})y_{10}+(k_{4}-k_{7}-k_{5}+k_{8})y_{20}}+1} \end{array}$$

Let $k_{1}e^{k_{5}}=t_{1},\;k_{2}e^{k_{8}}=t_{2},\;k_{3}-k_{5}=t_{3},\;k_{4}-k_{5}=t_{4},k_{6}-k_{8}=t_{5},\text{ and }k_{7}-k_{8}=t_{6}$ ; then, the final result is:

A-Eq. (8)
$$\{\begin{array}{l} \;y_{11}=\frac{1}{1+t_{1}e^{t_{3}y_{10}+t_{4}y_{20}}+t_{2}e^{t_{5}y_{10}+t_{6}y_{20}}}\\ y_{21}=\frac{1}{(1/t_{1})e^{-t_{3}y_{10}-t_{4}y_{20}}+1+(t_{2}/t_{1})e^{(t_{5}-t_{3})y_{10}+(t_{6}-t_{4})y_{20}}}\\ y_{31}=\frac{1}{(1/t_{2})e^{-t_{5}y_{10}-t_{6}y_{20}}+(t_{1}/t_{2})e^{(t_{3}-t_{5})y_{10}+(t_{4}-t_{6})y_{20}}+1} \end{array}$$

A-Eq. (8) is Eq. (1). The derivation shows that t_1_ and t_2_ must be greater than zero.
